# Optimization of the Sequential Extraction Process of Compounds for the Integral Valorization of *Macrocystis pyrifera*

**DOI:** 10.1021/acsomega.6c03104

**Published:** 2026-07-01

**Authors:** Francisca Crislândia Oliveira Silva, Giovanna de Farias Barreto, Thiago Barbosa Cahú, Francisco Felipe Bezerra, Lidilhone Hamerski, Javier Infante Rosselot, Mauro Sérgio Gonçalves Pavão

**Affiliations:** † Federal University of Rio de Janeiro, 28125 Institute of Medical Biochemistry, Laboratory of Biochemistry and Cell Biology of Glycoconjugates, Street Prof°. Rodolpho P. Rocco, 255, Rio de Janeiro 21941-913, Brazil; ‡ Federal University of Rio de Janeiro, Institute of Medical Biochemistry, Connective Tissue Laboratory, Street Prof°. Rodolpho P. Rocco, 255, Rio de Janeiro 21941-913, Brazil; § Federal University of Rio de Janeiro, Natural Products Research Institute, Carlos Chagas Filho Street 373, Rio de Janeiro 21941-902, Brazil; ∥ Ocean Rainforest, Inc., 1117 State Street, Santa Barbara, California 93101, United States

## Abstract

This study aimed to optimize the sequential extraction of high value-added compounds from the brown macroalga *Macrocystis pyrifera*. The biomass presented high carbohydrate (43.76%) and ash (36.20%) contents, with lower levels of proteins (9.11%), lipids (3.75%), and phenolic compounds (0.33%). Elemental analysis indicated carbon (38.75%) and oxygen (38.33%) as the predominant elements. Sequential extraction enabled the recovery of biostimulants (via osmotic and thermal treatments), fucoidan, and alginate. A factorial design established optimal extraction conditions at 60 °C, pH 3.5, and 1 h, based on chemical characterization parameters. Fucoidan exhibited a molecular weight of 447.61 kDa, while alginate showed 327.35 kDa. FTIR analysis revealed characteristic polysaccharide bands (O–H, C–O–C, and C–C). Monosaccharide analysis indicated that fucose and galactose were the main components of fucoidan, whereas alginate was composed of mannuronic (M) and guluronic (G) acids. Nuclear magnetic resonance (NMR) spectroscopy showed that fucoidan consists of a backbone of α-l-fucopyranose residues linked by (1→3) and (1→4) bonds, partially sulfated at C-2, with β-d-glucuronic acid and β-d-galactose residues, as well as terminal α-d-glucopyranose units. Alginate exhibited MM, GG, MG, and GM blocks, with predominance of MG [(→4)-β-d-Man*p*A-(1→4)-α-l-Gul*p*A-(1→)]. These results highlight the efficiency of the optimized process for biomass valorization and biotechnological applications.

## Graphical abstract

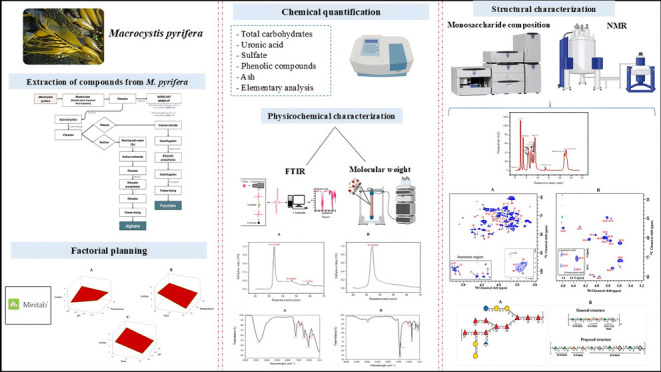

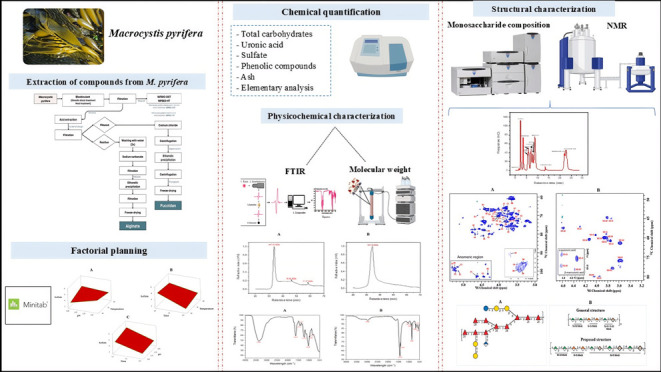

## 1 Introduction

Seaweeds are rich source of various bioactive compounds, including polysaccharides, minerals, vitamins, polyphenols, pigments, hormones, proteins, and lipids.^1^ *Macrocystis pyrifera* is a brown macroalga characterized by its rapid growth, diversity of bioactive compounds, and high biomass production rate, which makes it a strategic and promising raw material for the sustainable production of high value-added compounds.^2^

Biostimulants are one of the main value-added compounds derived from macroalgae, with the ability to promote plant growth, increase nutrient use efficiency, improve tolerance to abiotic stresses, and stimulate plant defense mechanisms.^3^ The effects of biostimulants are attributed to the presence of compounds such as amino acids, polyphenols, minerals, proteins, carbohydrates, and phytohormones, which act synergistically to modulate the physiological and metabolic processes of plants.^4,5^ In particular, extracts obtained by physical treatments such as osmotic shock and heat treatment have shown greater bioavailability of soluble compounds, favoring their action in plant tissues and justifying their use as the first step in sequential extraction processes.^4^

Polysaccharides represent about 30–60% of the dry mass of brown macroalgae, characterized by different monosaccharide compositions, types of glycosidic bonds, degrees of branching, and a wide distribution of molecular weights.^6,7^ Fucoidan is a sulfated heteropolysaccharide composed mainly of fucose units and smaller proportions of galactose, glucose, xylose, mannose, and uronic acids, linked by α-(1→3) or α-(1→4) glycosidic bonds. It is associated with multiple biological activities such as antioxidant, anti-inflammatory, anticoagulant, and immunomodulatory effects.^8^ Alginate is a linear anionic polysaccharide consisting of blocks of β-d-mannuronic (M) and α-l-guluronic (G) acids, arranged in homopolymeric and heteropolymeric sequences, with gelling, thickening, and stabilizing properties.^9^

Traditional methods for extracting compounds from macroalgae include acidic, alkaline (with and without heat), enzymatic and ultrasound-assisted treatment.^10–12^ Although effective, these methods are often applied in isolation, which can result in low biomass utilization, structural degradation of polymers, and excessive waste generation. In addition, harsh extraction conditions can compromise the chemical and functional integrity of polysaccharides.^13,14^ Thus, strategies based on biorefinery approaches that integrate different extraction steps in a rational and controlled manner, are essential to maximize the valorization of algal biomass.^14^

Therefore, the optimization of sequential extraction processes has emerged as a promising approach for the full utilization of *M. pyrifera*, allowing the orderly recovery of biostimulants, fucoidans, and alginates from the same biological matrix.^15^ This strategy not only maximizes the overall yield of the extracted compounds, but also controls the chemical and structural characteristics of each fraction, reducing losses and promoting greater sustainability in the process.^16^

In this context, this study aimed to optimize the sequential extraction process of compounds from *M. pyrifera* with the initial goal of obtaining biostimulants through physico chemical treatments, followed by the recovery of fucoidan and alginate from the remaining solid residue. This approach represents an integrated and sustainable strategy for the valorization of brown macroalga biomass, contributing to the development of more efficient, economically viable, and environmentally sustainable processes.

## 2 Material and Methods

### 2.1 Biological Material

The brown macroalga *M. pyrifera* was collected by Ocean Rainforest Incorporation (California, USA) in the Faroe Islands – Denmark (62° 00′ N, 7° 00′ W) during winter and taxonomically identified by a specialist. The fresh biomass was washed with distilled water to remove salts and surface impurities, dried in a forced-air oven at 55 ± 2 °C until constant weight, and subsequently milled into a fine powder using a mechanical blender (Moulinex, France). The processed material was provided by Ocean Rainforest Incorporation for experimental purposes.

### 2.2 Chemical Quantification and Elemental Analysis of *M. pyrifera* Biomass

The biomass of *M. pyrifera* was chemically characterized. The moisture and ash contents were determined by thermogravimetric analysis (TGA) with programmed heating up to 800 °C, allowing the quantification of mass losses associated with water evaporation and organic matter decomposition.^17^ Protein content was quantified by the Kjeldahl method, based on total nitrogen determination using a nitrogen digester and distiller. The nitrogen-to-protein conversion factor of 5.0 was applied to estimate crude protein content.^18^ Total phenolic compounds were determined by spectrophotometric method, using the Folin–Ciocalteu reagent.^19^ Lipids were determined by Soxhlet extraction, followed by solvent evaporation and residue quantification,^20^ whereas the total carbohydrate content was estimated by mass difference, considering the remaining balance after the quantification of the other constituent fractions of the biomass.^21^

For elemental analysis, approximately 0.2 g of dry *M. pyrifera* biomass was weighed using a high-precision microbalance. The levels of carbon (C), hydrogen (H), nitrogen (N), sulfur (S), and oxygen (the latter estimated by mass difference) were determined using a PerkinElmer CHNS/O 2400 Series II elemental analyzer.^22^

### 2.3 Extraction of Compounds from *M. pyrifera*

#### 2.3.1 Extraction of Biostimulant

##### 2.3.1.1 Osmotic Shock Treatment

*M. pyrifera* biostimulant was obtained by the osmotic shock method, as described by Postma et al.,^23^ with some modifications. Initially, the dry biomass was crushed in an industrial cutting grinder (KD Eletro LAR-2, São Paulo, Brazil) and suspended in distilled water at a ratio of 1% (w/v). The suspension was kept under constant magnetically stirring (200 rpm) for 24 h at room temperature (25 °C) to promote the diffusion of water-soluble constituents into the extracting medium. The mixture was then filtered through a nylon mesh (300 mesh) to remove solid residues (used for polysaccharide extraction), and the filtrate obtained was collected, subjected to lyophilization, and the final yield was determined, being named *M. pyrifera* biostimulant obtained by osmotic shock treatment (MPBIO-OST).

##### 2.3.1.2 Heat Treatment

*M. pyrifera* biostimulant was obtained by the high-temperature aqueous extraction method described by Han et al.,^24^ with some modifications. The biomass was initially crushed in an industrial cutting grinder (KD Eletro LAR-2, São Paulo, Brazil) and suspended in distilled water at a concentration of 1% (w/v). The suspension was kept under magnetically stirring (200 rpm) and boiled at 100 °C for 30 min to increase the solubilization of structural components. After heat treatment, the mixture was filtered through a nylon mesh (300 mesh) to separate the solid residues, and the resulting filtrate was collected, subjected to lyophilization, and the yield determined, being named *M. pyrifera* biostimulant obtained by heat treatment (MPBIO-HT).

#### 2.3.2 Extraction of Polysaccharides

##### 2.3.2.1 Factor Planning

The extraction of the polysaccharides (fucoidan and alginate) from *M. pyrifera* was based on a factorial design aimed at analyzing the influence of yields and chemical composition on the independent variables (Table S1; Supporting Information). These variables included temperature, pH and extraction time. The methodology was followed according to.^25,26^ In this plan, the actual values of each factor were coded as +1 and −1, representing levels above and below the midpoint, respectively. The coding of level values is presented in Tables S1 and S2.

After assigning codes to the levels of each factor to create specific combinations used in the factorial design, 8 unique combinations were obtained (Table 1). These combinations were designated as extraction 1, 2, 3, 4, 5, 6, 7, and 8. Each combination varies in terms of the levels of each factor, allowing the study to assess the impact of these variations on response variables, such as yield and chemical composition. The statistical software Minitab 18 (Minitab Inc., Pennsylvania, USA) was used to help make the matrix.

**Table 1:** Factorial Planning Matrix of Extraction Composition

**Extraction**	**Temperature (°C)**	**pH**	**Time (hours)**
1	60	3.0	1
2	60	3.0	2
3	60	3.5	1
4	60	3.5	2
5	80	3.0	1
6	80	3.0	2
7	80	3.5	1
8	80	3.5	2

##### 2.3.2.2 Extraction of Fucoidan and Alginate

After obtaining the biostimulant by osmotic shock, the remaining residue was extracted with 0.05 M HCl, using a solid-to-liquid ratio of 4.5% (m/v), corresponding to the proportion between the residue and the HCl solution (1:20). The pH, temperature, and extraction time were established according to the factorial design, under magnetic stirring (200 rpm). After the acid extraction, the solution was filtered through a nylon filter (300 mesh). The residue was reserved for alginate extraction and the filtrate used for obtaining fucoidan.^27^ The residue was washed twice with distilled water to remove the acid and extracted with Na_2_CO_3_ (4.5% m/v) at 45 °C for 2 h, under constant agitation (200 rpm). The alginate rich solution was then precipitated with 80% (v/v) ethanol, centrifuged (3000 rpm/10 min), lyophilized and ground using a knife mill to obtain alginate (ALG). For the extraction of fucoidan, the filtrate was treated with CaCl_2_ (3%) to remove the crude alginate, centrifuged (3000 rpm,10 min) and the supernatant precipitated with 80% (v/v) ethanol. The precipitate formed was collected by centrifugation (3000 rpm, 10 min) and lyophilized to obtain fucoidan (FUC) (Figure 1).

**Figure 1:**
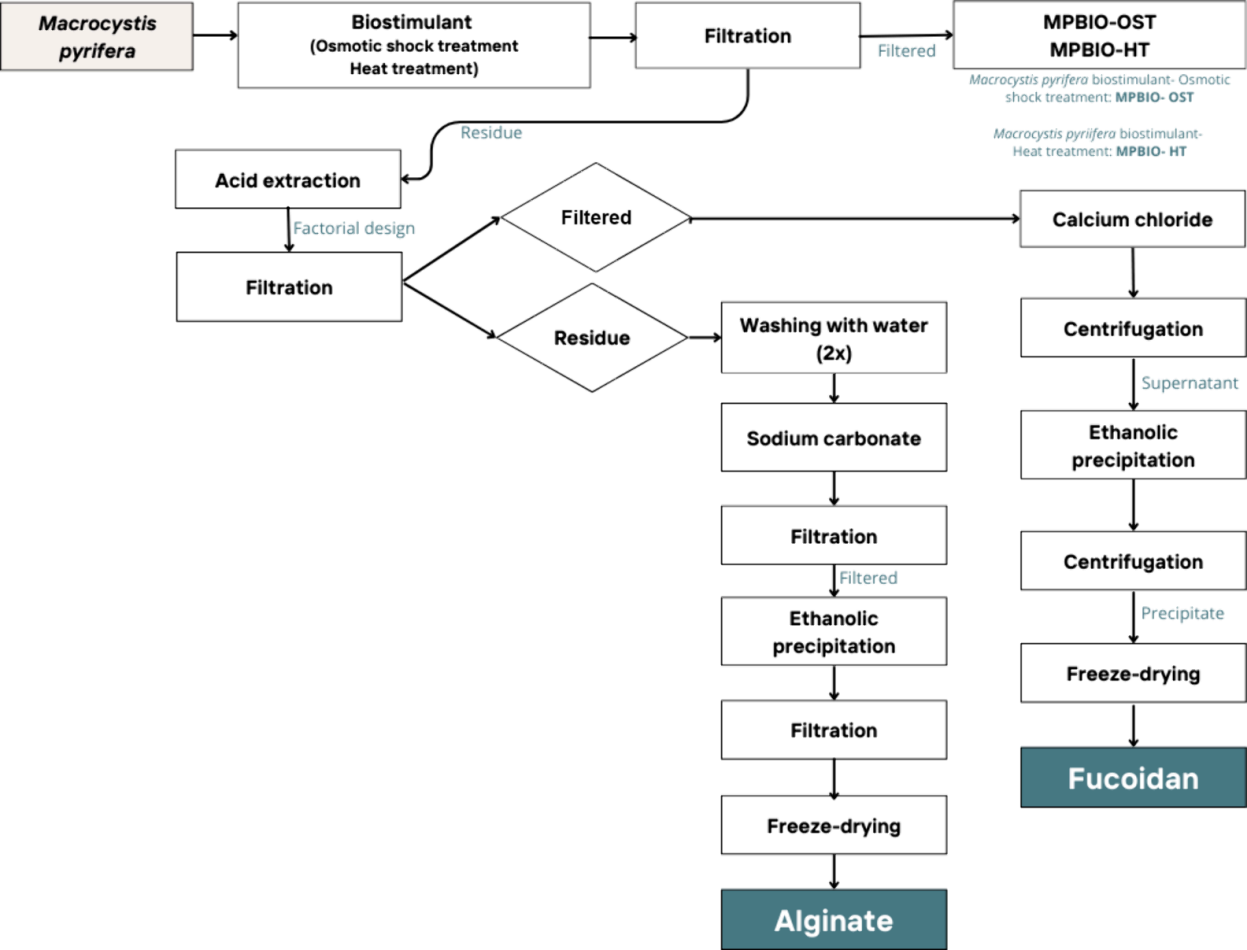
Representation of the sequential extraction process of the biostimulant, fucoidan and alginate from *M. pyrifera*. Description: *M. pyrifera* biostimulant obtained by osmotic shock treatment (MPBIO-OST); *M. pyrifera* biostimulant obtained by heat treatment (MPBIO-HT).

### 2.4 Chemical Characterization

*M. pyrifera* biostimulants were quantified in terms of total carbohydrate, protein, phenolic compound, and lipid contents, whereas the extracted polysaccharides, namely fucoidan and alginate, were characterized with respect to their carbohydrate, hexuronic acid content, and sulfate contents.

Spectrophotometric analyses were employed to determine the total carbohydrate content (Dubois et al.,^28^), hexuronic acid (Dische,^29^), sulfate content (Dodgson and Price,^30^), total protein (Bradford,^31^), and phenolic compound content (Singleton and Ross,^19^) of *M. pyrifera* compounds. Fucose (A490 nm), α-glucuronolactone (A525 nm), anhydrous sodium sulfate (A360 nm), bovine serum albumin (A595 nm), and gallic acid (A750 nm) were used as the analytical standards, respectively. Lipids were determined by Soxhlet extraction, solvent evaporation and gravimetric quantification of the residue.^20^

The elemental composition of the biostimulants was determined by inductively coupled plasma optical emission spectrometry (ICP-OES) following acid digestion performed at Solotech Cerrado (Goiás, Brazil). An aliquot of 25 mL of sample was digested with 5 mL of concentrated nitric acid (HNO_3_, 65%, v/v) under controlled heating at 120 °C for 2 h. After cooling, the digested solution was diluted to a final volume of 40 mL with ultrapure water. The concentrations of macronutrients (nitrogen (N), phosphorus (P), potassium (K), calcium (Ca), magnesium (Mg), and sulfur (S)) and micronutrients (iron (Fe), manganese (Mn), copper (Cu), zinc (Zn), and boron (B)) were quantified by external calibration with certified standard solutions.^32^

### 2.5 Estimation of Molecular Mass

The molecular weight of fucoidan and alginate was estimated by High Performance Size Exclusion Chromatography (HPSEC) using a Shimadzu HPLC system equipped with a pump (LC-20AT), differential refractive index detector (RID-10A), and UV/vis detector (SPD-20AV). Separations were performed using a Superose 6 column (300 mm × 10 mm; Pharmacia Fine Chemicals). The mobile phase consisted of an aqueous buffer (pH 7.5) containing TRIS-HCl (20 mM), EDTA (1 mM), and NaCl (150 mM). The mobile phase flow rate was 0.5 mL/min. The samples were dissolved in the mobile phase (5 mg/mL) and injected manually (20 μL). The system was calibrated using dextran standards of different molecular weights (5, 12, 25, 50, 70, 80, 270, and 670 kDa). A third-order polynomial fitted to the calibration results (logM versus retention time) was used to estimate the molecular weight of fucoidan and alginate.^33^

### 2.6 Physical–Chemical Characterization by Fourier Transform Infrared Spectroscopy (FTIR)

The Fourier Transform Infrared (FTIR) spectroscopy analysis of fucoidan and alginate (10 mg) of *M. pyrifera* (extraction method 3) was performed in an ATR (Attenuated Total Reflection) window and analyzed in the wavelength range from 500 to 4000 cm^–1^ with a resolution of 4 cm-^1^ and an average measurement of 120 scans (PerkinElmer Spectrophotometer) (Silva et al., 2025). The vibration spectra were collected and analyzed using an Essential FTIR 1.1.0.0 Software (Madison, USA) and Sigma Plot software version 12.0 (Systat Software, Inc., California, USA).^34^

### 2.7 Monosaccharide Composition

A two-step hydrolysis with sulfuric acid was performed to analyze the monosaccharide composition of fucoidan and alginate extracted from *M. pyrifera*. 10 mg/mL of the polysaccharides were mixed with 72% w/w H_2_SO_4_ and incubated at 30 °C for 1 h. The reactions were then diluted to a final acid concentration of 4% (w/w) H_2_SO_4_, and hydrolysis was continued at 100 °C for 3 h in a dry bath. Acid hydrolysates were dried on a rotary evaporator and diluted with water prior to monomer analysis. Analysis of the monosaccharide composition was carried out by high performance anion exchange chromatography with pulsed amperometric detection (HPAEC-PAD) using an ICS5000+ system (Dionex, Sunnyvale, USA) on a CarboPac PA1 column (4 × 250 mm) and guard column (2 × 50 mm). Neutral sugars were eluted with a linear gradient of 100 mM NaOH and uronic acids were eluted isocratically with sodium acetate (100 mM). Quantification was carried out using Origin software. The recovery values for the monosaccharides were estimated from parallel runs of acid hydrolysis of the monosaccharide standards: mannitol, fucose, arabinose, rhamnose, galactose, glucose, xylose, mannose, guluronic acid, mannuronic acid and glucuronic acid.^27,35^

### 2.8 Structural Characterization by Nuclear Magnetic Resonance (NMR)

The NMR spectra of fucoidan and alginate were recorded using a 600 MHz Bruker DRX spectrometer with a triple resonance probe at 60 °C, as described by de Araujo et al.^36^ Approximately 10 mg of sample was dissolved in 0.55 mL of 99.6% deuterium oxide (D_2_O), which was used as the solvent. Chemical shifts were referenced to tetramethylsilane (TMS, 0.00 ppm). Water signal suppression was achieved by presaturation. The ^1^H NMR spectrum of fucoidan was recorded using 16 scans. The Heteronuclear Single Quantum Correlation Edited Spectroscopy (HSQC) spectrum of ^1^H–^13^C (1024 × 256 points) of fucoidan and alginate were acquired with alternating rectangular phase pulses, globally optimized for decoupling. The ^1^H and ^13^C chemical shifts were calibrated based on the signals of trimethylsilylpropionic acid as a standard at 0 ppm. The spectra were processed using Top-Spin 3.6.5 software (Bruker).

### 2.9 Statistical Analysis

All experimental procedures were performed in triplicate and results were expressed as mean ± standard deviation. Data were analyzed using three-way analysis of variance (three-way ANOVA) to evaluate the effects of the independent factors, their interactions and experimental trials. When significant differences were detected, Tukey’s post hoc test was applied for multiple comparisons. Statistical analyses were performed using GraphPad Prism version 8.0 (GraphPad Software Inc., California, USA) and Minitab, with significance set at *p* < 0.05. Different letters indicate statistically significant differences among experimental groups.

## 3 Results and Discussion

### 3.1 Biomass Composition of *M. pyrifera*

The centesimal composition of *M. pyrifera* biomass showed a chemical profile typical of brown macroalgae (Figure 2A). Carbohydrates constituted the main fraction of the biomass, corresponding to 43.76%, followed by ash content, which represented 36.20%. The residual moisture was 6.83%, while lipid and protein/nitrogen fractions accounted for 3.75 and 9.11%, respectively. Total phenolic compounds corresponded to 0.33% of the total composition. Elemental analysis of *M. pyrifera* biomass determined its elemental stoichiometry, revealing the predominance of carbon (38.75%) and oxygen (38.33%), followed by nitrogen (14.28%), hydrogen (6.31%), and sulfur (2.32%) (Figure 2B).

**Figure 2:**
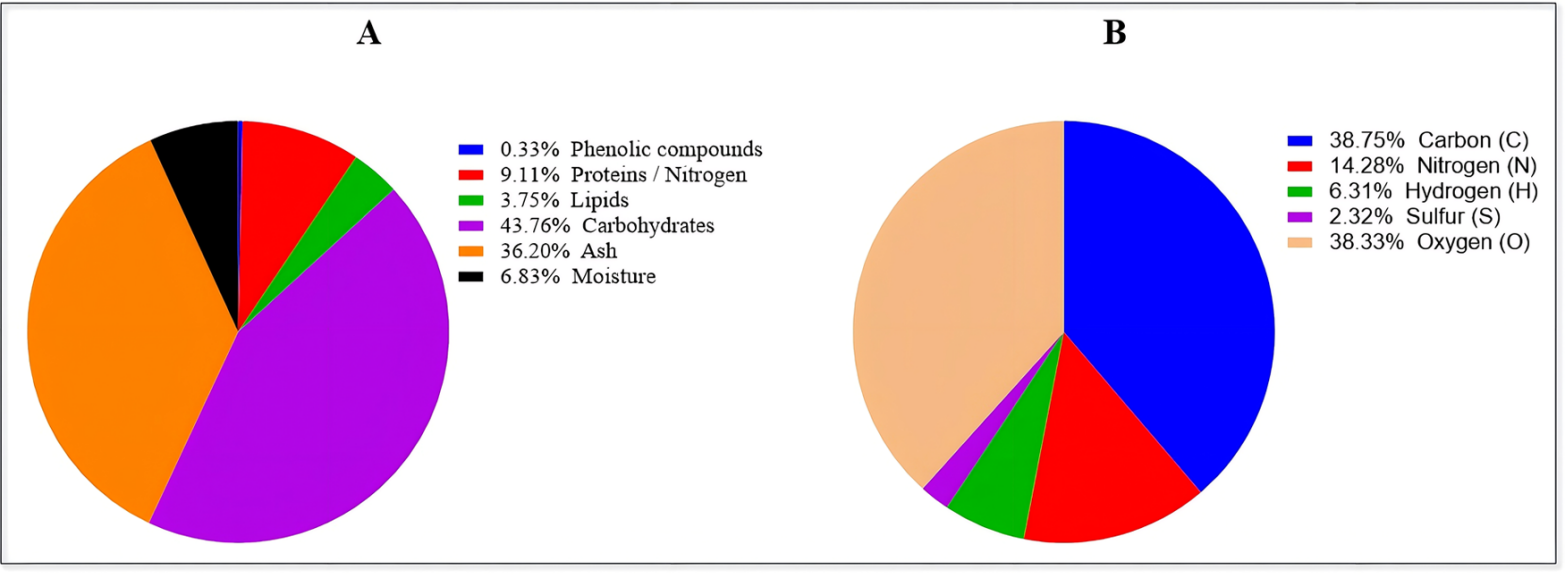
Biomass composition of *M. pyrifera*: (A) centesimal composition and (B) elemental analysis.

The high carbohydrate content reflects the presence of structural and reserve polysaccharides characteristic of *M. pyrifera*, such as fucoidans, alginates, and laminaranes, widely described as the main constituents of the cell wall and extracellular matrix of this macroalgae.^6^ The high ash content is associated with this species’ significant capacity to accumulate minerals from the marine environment, often related to the significant presence of ions such as calcium, magnesium, potassium, and sodium, in addition to other trace elements incorporated into the polysaccharide matrix. Low residual moisture indicates adequate biomass drying conditions, contributing to greater material stability during storage and subsequent analyses.^22^ The lipid content was relatively low, whereas the protein fraction represented a moderate proportion of the biomass, remaining within the range commonly reported for brown macroalgae.^37,38^ The low concentration of phenolic compounds suggests that, although biologically relevant, these metabolites occur in lower proportions when compared to structural polysaccharides and minerals present in algal biomass.^39^

The high oxygen and carbon contents are directly related to the abundance of structural and reserve polysaccharides, such as alginates, fucoidans, and laminarans, whose chains are rich in hydroxyl, carboxyl, and sulfate groups, contributing substantially to the elemental composition of the biomass. Carbon represents the organic backbone of these biopolymers, forming the structural basis of the algal cell wall and extracellular matrix.^40^ Nitrogen accounted for 14.28% of the elemental composition, indicating the presence of nitrogen-containing compounds in the biomass, while sulfur (2.32%), although present in lower proportions compared to carbon and oxygen, is associated with sulfate groups of sulfated heteropolysaccharides such as fucoidans.^41^

### 3.2 Yield and Chemical Characterization of Biostimulants

The yield and chemical composition of the biostimulants obtained from *M. pyrifera* biomass varied significantly depending on the extraction method used (Table 2). The biostimulant obtained by heat treatment (MPBIO-HT) showed a significantly higher yield (18.00 ± 1.414%) when compared to the biostimulant obtained by osmotic shock (MPBIO-OST), which had a yield of 6.50 ± 6.363% (*p* < 0.05). This increase in yield indicates that heating favors the solubilization and release of water-soluble compounds from the algal matrix, promoting the extraction of bioactive constituents.^41^

**Table 2:** Yield and Chemical Characterization of the Biostimulants: *M. pyrifera* Biostimulant Obtained by Osmotic Shock Treatment (MPBIO-OST) and *M. pyrifera* Biostimulant Obtained by Heat Treatment (MPBIO-HT)^a^

**Analyses**	**MPBIO-OST**	**MPBIO-HT**
Yield (% dry mass)	6.50 ± 0.363^a^	18.00 ± 1.414^b^
Carbohydrate (mg of hexose/g of sample)	7.56 ± 0.890^a^	23.41 ± 0.51^b^
Proteins (% dry mass)	1.295 ± 0.106^a^	0.345 ± 0.106^b^
Phenolic compounds (mg gallic acid/g of sample)	0.53 ± 0.107^a^	0.32 ± 0.110^b^
Lipids (% dry mass)	2.55 ± 0.636	2.05 ± 0.251

a Different letters indicate statistically significant differences between the biostimulant extracts. Differences were considered statistically significant at *p* < 0.05.

Regarding carbohydrate content, a statistically significant difference was observed between the methods, with MPBIO-HT presenting a concentration approximately three times higher (23.41 ± 0.51 mg of hexose/g of sample) compared to MPBIO-OST (7.56 ± 0.890 mg of hexose/g of sample). The protein content was higher in the biostimulant obtained by osmotic shock (1.295 ± 0.106%) compared to MPBIO-HT (0.345 ± 0.106%), indicating that heating can promote denaturation or partial degradation of proteins, reducing their recovery in the final extract. Similar behavior was observed for total phenolic compounds, whose content was significantly higher in MPBIO-OST (0.53 ± 0.107 mg gallic acid/g sample) when compared to MPBIO-HT (0.32 ± 0.110 mg gallic acid/g sample). In addition, no significant differences were observed in lipid concentration between the treatments (*p* > 0.05), with low levels detected in both biostimulants, presenting values of 2.55 ± 0.636% for MPBIO-OST and 2.05 ± 0.251% for MPBIO-HT, which is consistent with the typically low lipid profile of brown macroalgae (Table 2). These results suggest that osmotic shock preserves heat-sensitive compounds such as phenolics and proteins.^42^

In addition, an elemental characterization of the macronutrients and micronutrients in the biostimulants obtained by different extraction methods was performed (Figure 3; Table S3, Supporting Information). Both extracts contained significant concentrations of macronutrients, particularly potassium, calcium, and magnesium. Nitrogen levels were identical in both biostimulants (190 mg/L). Potassium showed high values in both treatments, with 1340 mg/L for MPBIO-OST and 1350 mg/L for MPBIO-HT. In contrast, the biostimulant obtained by osmotic shock (MPBIO-OST) exhibited higher concentrations of calcium (470 mg/L) and magnesium (350 mg/L) compared to MPBIO-HT (430 mg/L and 330 mg/L, respectively), and a significantly higher sulfur content in the form of sulfate (190 mg/L in MPBIO-OST and 140 mg/L in MPBIO-HT). Regarding micronutrients, the biostimulants exhibited detectable concentrations of iron, manganese, copper, zinc, and boron. Iron levels were identical in both extracts (0.53 mg/L), while those of manganese (0.06 vs 0.04 mg/L), copper (0.14 vs 0.10 mg/L), zinc (0.03 vs 0.01 mg/L) were higher in MPBIO-OST compared to MPBIO-HT, with a significant difference in boron content (6.10 vs 5.14 mg/L), indicating better preservation or solubilization of these nutrients under osmotic shock extraction conditions.

**Figure 3:**
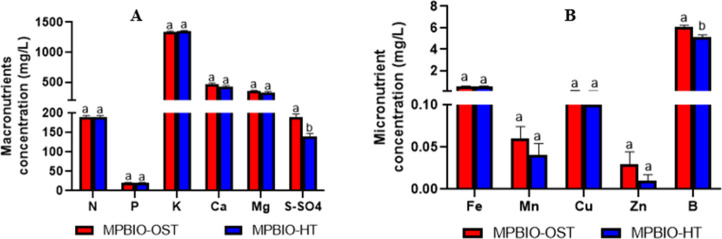
Elemental analysis of macronutrients and micronutrients of biostimulants: *M. pyrifera* biostimulant obtained by osmotic shock treatment (MPBIO-OST) and *M. pyrifera* biostimulant obtained by heat treatment (MPBIO-HT). Macronutrients: nitrogen (N), phosphorus (P), potassium (K), calcium (Ca), magnesium (Mg), and sulfur (S). Micronutrients: iron (Fe), zinc (Zn), copper (Cu), and manganese (Mn). Different letters indicate statistically significant differences between the biostimulant extracts. Differences were considered statistically significant at *p* < 0.05.

Biostimulants promote plant growth and development throughout the crop cycle by improving plant metabolism efficiency, production quality, and resilience to abiotic and biotic stresses.^3^ In this context, the presence of macronutrients such as nitrogen (N), phosphorus (P), and potassium (K) is essential for the synthesis of biomolecules, expansion, and differentiation of plant tissues, while calcium (Ca) and magnesium (Mg) act in metabolic signaling and photosynthetic processes, including enzyme activation and chlorophyll formation. The presence of sulfur in the form of sulfate (S–SO_4_^2–^) acts directly on the biosynthesis of amino acids and proteins.^43^ In addition, the balanced composition of micronutrients observed in these biostimulants also suggests a synergistic effect on the absorption and efficient use of nutrients, acting as enzymatic cofactors and regulators of physiological processes, which can result in the optimization of plant physiological performance.^44^

Based on comparative chemical and elementary characterization, the biostimulant obtained by osmotic shock treatment (MPBIO-OST) was selected for the continuation of the sequential extraction process aimed at obtaining fucoidans and alginates. This choice is based on the fact that osmotic shock treatment promotes the preferential extraction of water-soluble and metabolically active fractions, preserving structural components of the cell wall.^10,23^ In addition, MPBIO-OST had higher levels of proteins, phenolic compounds, macronutrients, and mineral micronutrients when compared to the biostimulant obtained by heat treatment, indicating a potentially more bioactive chemical profile and less thermal degradation of macromolecules. The higher concentration of these constituents is particularly relevant from a physiological point of view, since proteins, phenolics, and minerals act synergistically in modulating plant metabolic processes, including growth, nutrient absorption, and responses to abiotic stresses.^3,44^

### 3.3 Yield and Chemical Characterization of Fucoidan and Alginate

The fucoidan extraction yields varied significantly among the different conditions established in the factorial design (Table 3). Among all combinations of temperature, pH, and time, the highest yields were observed in extractions 5 (3.26 ± 0.021%) and extraction 3 (3.15 ± 0.054%), which did not show a statistical difference between them (*p* > 0.05), indicating equivalent efficiency in fucoidan recovery. Although these two operating conditions differed in the experimental parameters, with extraction 5 performed at 80 °C, pH 3.0 for 1 h, and extraction 3 conducted at 60 °C, pH 3.5 for 1 h., the results suggest that the reduced time (1 h) had a greater influence on fucoidan yield than temperature and pH factors. In contrast, the conditions that employed 2 h of extraction presented lower yields, suggesting that prolonged exposure of the biomass to the acidic medium and high temperature may have promoted degradation, excessive solubilization of nonpolysaccharide components, or loss of structural fractions, reducing fucoidan recovery.^6^

**Table 3:** Yield and Chemical Characterization of Fucoidan Extracted from *M. pyrifera* ^a^

**Extraction**	**Yield (% dry mass)**	**Carbohydrate (mg of hexose/g of sample)**	**Hexuronic acid (mg of hex. acid/g of sample)**	**Sulfate (mg of sulfate/g of sample)**
1	2.75 ± 0.025^a^	8.25 ± 0.142^a^	0.50 ± 0.026^a^	4.73 ± 0.075^a^
2	1.32 ± 0.047^b^	4.08 ± 0.097^b^	0.36 ± 0.032^a,b^	1.71 ± 0.077^b^
3	3.15 ± 0.054^c^	13.20 ± 0.047^c^	0.57 ± 0.047^a,c^	7.51 ± 0.062^c^
4	1.65 ± 0.064^d^	6.15 ± 0.021^d^	0.15 ± 0.034^d^	3.22 ± 0.028^d^
5	3.26 ± 0.021^c,e^	10.08 ± 0.142^e^	0.89 ± 0.022^e^	5.91 ± 0.026^e^
6	1.78 ± 0.024^d,f^	4.86 ± 0.123^f^	1.97 ± 0.029^f^	3.97 ± 0.054^f^
7	2.60 ± 0.014^g^	5.68 ± 0.045^g^	0.95 ± 0.074^e,g^	5.06 ± 0.064^g^
8	1.48 ± 0.005^h^	4.19 ± 0.045^b,h^	0.41 ± 0.086^a,b,c,h^	3.45 ± 0.059^d,h^

a Different letters indicate a statistically significant difference between fucoidan extractions. Comparisons were considered significant for *p* < 0.05.

The total carbohydrate content showed strong sensitivity to extraction conditions (Table 3). The highest carbohydrate content was found in extraction 3 (60 °C; pH 3.5; 1 h), with 13.20 ± 0.047 mg of hexose/g of sample, a value significantly higher than all other conditions (*p* < 0.05). This indicates that moderate temperature and less acidic pH, associated with a shorter extraction time, favor the maintenance of the integrity and solubilization of polysaccharides. In contrast, extractions performed at pH 3.0 and for 2 h (extractions 2 and 6) had the lowest carbohydrate contents (4.08 ± 0.097 and 4.86 ± 0.123 mg/g, respectively), suggesting possible degradation or loss of polysaccharide fractions under higher conditions.^45^

The hexuronic acid contents ranged from 0.15 ± 0.034 to 1.97 ± 0.029 mg/g, indicating that the extraction conditions significantly affect the recovery of these fractions (*p* < 0.05) (Table 3). The highest hexuronic acid value was obtained in extraction 6 (80 °C; pH 3.0; 2 h), while extraction 4 (60 °C; pH 3.5; 2 h) had the lowest hexuronic acid content (0.15 mg/g), ensuring that high temperatures and prolonged times favored the release of uronic acids, possibly due to greater disruption of the structural matrices.^27^

It is important to note that high levels of hexuronic acids in fucoidan preparations are generally indicative of copurification of unsulfated acidic polysaccharides, such as alginates and hemicelluloses, which make up the structural matrix of macroalgae. These components can be solubilized under higher extraction conditions, especially when high temperatures and prolonged times are used.^8,46^ In this context, the higher concentrations of hexuronic acid observed in extractions conducted at higher temperatures (extractions 5, 6, and 7) reinforce this trend, suggesting greater coextraction of uronic fractions.

The sulfate content also varied significantly among the conditions tested (*p* < 0.05) (Table 3). The highest sulfate concentration was observed in extraction 3 (7.51 ± 0.062 mg/g), the same condition that presented the highest carbohydrate content, suggesting that pH 3.5 and 60 °C favor the extraction of more sulfated fucoidans with low hexuronic acid content. Extractions 2 and 4 had the lowest sulfate contents (1.71 ± 0.077 and 3.22 ± 0.028 mg/g), both performed at pH 3.0, indicating that more acidic pH reduces the recovery of sulfated fractions. At 80 °C, an intermediate trend was observed, with levels ranging from 3.97 to 5.90 mg/g. The data show that pH may be the main factor modulating sulfate concentration, with moderately acidic conditions (around pH 3.5) associated with lower temperatures and reduced time promoting greater release of sulfated polysaccharides, while more acidic conditions reduce the recovery of sulfate-rich fractions.^47,48^

The sulfate concentration is particularly important for determining the best method for extracting fucoidan, since this parameter is directly related to its structural and functional properties. In this context, based on factorial planning, the response surfaces of the sulfate content show main effects and significant interactions between temperature, pH, and extraction time (Figure 4). The temperature × pH interaction (Figure 4A) indicates that higher sulfate values are obtained at moderate acid pH (pH 3.5), especially when associated with low temperature (60 °C), showing that less severe conditions favor the preservation of sulfate groups in the fucoidan structure. Consistently, the temperature × time (Figure 4B) and pH × time (Figure 4C) surfaces show that shorter extraction times (1 h), combined with milder temperatures, result in higher sulfate contents, while the simultaneous increase in temperature and time tends to reduce this variable, possibly due to partial desulfation or structural degradation processes. These results corroborate the quantitative data in Table 3, in which the condition of 60 °C, pH 3.5, and 1 h presented the highest sulfate content, indicating that the factorial design was effective in identifying optimal conditions that maximize fucoidan sulfation.

**Figure 4:**
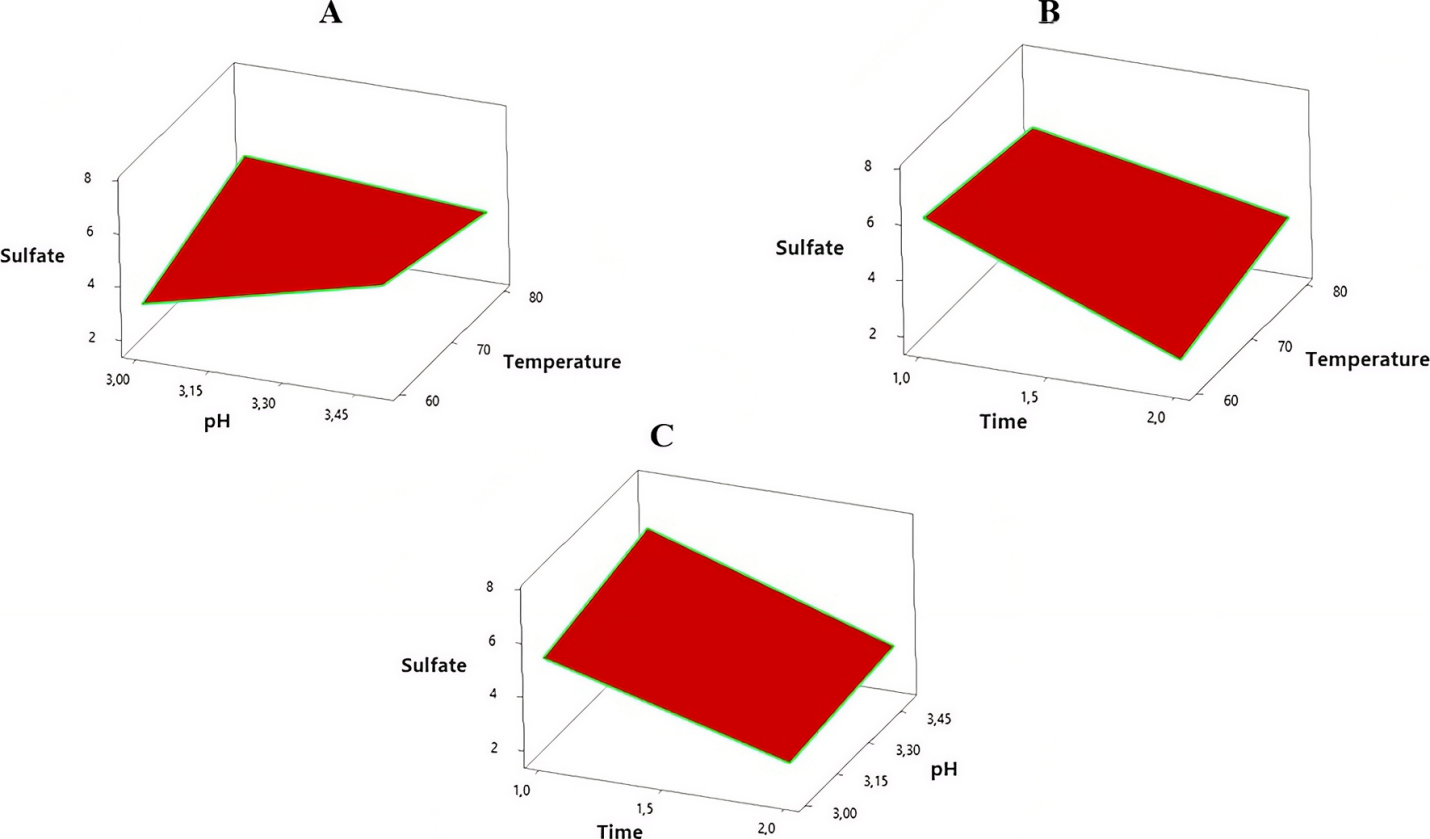
Response surface plot of the sulfate variable of fucoidan extracted from *M. pyrifera*: (A) temperature × pH, (B) temperature × time, and (C) pH × time.

The yields and chemical characterization of alginate extracted from *M. pyrifera* were also significantly influenced by the operating conditions established in the factorial design (Table 4) (*p* < 0.05). Yields ranged from 23.78 ± 0.005% to 36.63 ± 0.123% (dry weight), with the highest yield observed in extraction 4 (60 °C; pH 3.5; 2 h). Extractions 2 (32.33 ± 0.139%), 3 (34.34 ± 0.085%), and 5 (35.06 ± 0.184%) also showed high and statistically significant yields, with partially similar operating parameters. However, extraction 3 (60 °C; pH 3.5; 1 h), despite not presenting the highest absolute yield, stood out for combining a high yield with a chemical profile for high-purity alginates.

**Table 4:** Yield and Chemical Characterization of Alginate Extracted from *M. pyrifera* ^a^

**Extraction**	**Yield (% dry mass)**	**Carbohydrate (mg of hexose/g of sample)**	**Hexuronic acid (mg of hex. acid/g of sample)**	**Sulfate (mg of sulfate/g of sample)**
1	23.78 ± 0.005^a^	67.34 ± 0.074^a^	50.51 ± 0.113^a^	0.80 ± 0.004^a^
2	32.33 ± 0.139^b^	110.31 ± 0.124^b^	63.87 ± 0.118^b^	1.46 ± 0.007^b^
3	34.34 ± 0.085^c^	105.94 ± 0.078^c^	63.56 ± 0.147^b,c^	1.23 ± 0.024^c^
4	36.63 ± 0.123^d^	118.02 ± 0.094^d^	62.29 ± 0.102^d^	2.78 ± 0.022^d^
5	35.06 ± 0.184^e^	104.95 ± 0.075^e^	58.65 ± 0.115^e^	2.58 ± 0.024^e^
6	35.88 ± 0.163^f^	99.41 ± 0.162^f^	44.89 ± 0.142^f^	2.47 ± 0.027^e,f^
7	33.43 ± 0.121^e,f,g^	77.04 ± 0.113^g^	47.41 ± 0.135^g^	2.42 ± 0.075^f,g^
8	32.30 ± 0.139^b,h^	60.82 ± 0.112^h^	40.54 ± 0.133^h^	7.87 ± 0.064^h^

a Different letters indicate a statistically significant difference between alginate extractions. Comparisons were considered significant for *p* < 0.05.

Total carbohydrate contents showed wide variation between extraction conditions, ranging from 60.82 ± 0.112 mg/g to 118.02 ± 0.094 mg/g (*p* < 0.05) (Table 4). The highest concentrations were observed in extractions 4 (118.02 ± 0.094 mg/g), 2 (110.31 ± 0.124 mg/g), and 3 (105.94 ± 0.078 mg/g), suggesting that moderately acidic pH conditions associated with intermediate or high temperatures favor the solubilization of structural polysaccharides in the cell wall without promoting significant degradation of alginic chains.^47^ In addition, the hexuronic acid content varied significantly between conditions, ranging from 40.54 ± 0.133 mg/g to 63.87 ± 0.118 mg/g (*p* < 0.05). The highest levels were observed in extractions 2 (63.87 ± 0.118 mg/g), 4 (62.29 ± 0.102 mg/g), and 3 (63.56 ± 0.147 mg/g).

Extraction 3 showed a high total carbohydrate content (105.94 ± 0.078 mg hexose/g sample) and high hexuronic acid content (63.56 ± 0.147 mg/g), values statistically comparable to those obtained under conditions of higher yield (extractions 2, 4, and 5), indicating efficient solubilization of alginic chains rich in manuronic and guluronic acid units. These results suggest that the combination of moderate temperature, less acidic pH, and shorter extraction time favors the structural preservation of alginate, preventing excessive degradation of polymer chains.^49^

The sulfate content showed a wide variation, ranging from 0.80 ± 0.004 mg/g to 7.87 ± 0.064 mg/g (*p* < 0.05) (Table 4). The highest concentrations were found in extraction 8 (7.87 ± 0.064 mg/g) and extraction 4 (2.78 ± 0.022 mg/g). On the other hand, extractions 1, 3, and 2 had the lowest sulfate contents (0.80 ± 0.004, 1.23 ± 0.024, and 1.46 ± 0.007 mg/g, respectively), showing that milder conditions result in more selective extracts.^47^

### 3.4 Physicochemical and Structural Characterization of Fucoidan and Alginate

Polysaccharides are polydisperse polymers characterized by distributions of chains that share similar chemical structures but vary in length and, consequently, in molecular weight.^34^ Analysis of the molecular weight distribution profile of polysaccharides extracted from *M. pyrifera*, performed by HPSEC, revealed distinct behaviors for fucoidan and alginate. Fucoidan presented three peaks of lower intensity and resolution, reflecting a more heterogeneous character of the sample, while alginate exhibited a single well-defined peak, suggesting a relatively homogeneous molecular fraction (Figure 5A,B). Based on the calibration curve constructed from dextran standards (Figure S1: Supporting Information), the peak molecular weight (Mp) was estimated at approximately >447.61 kDa for fucoidan and >327.35 kDa for alginate, with the respective retention times and log values shown in Table S4 (Supporting Information).

**Figure 5:**
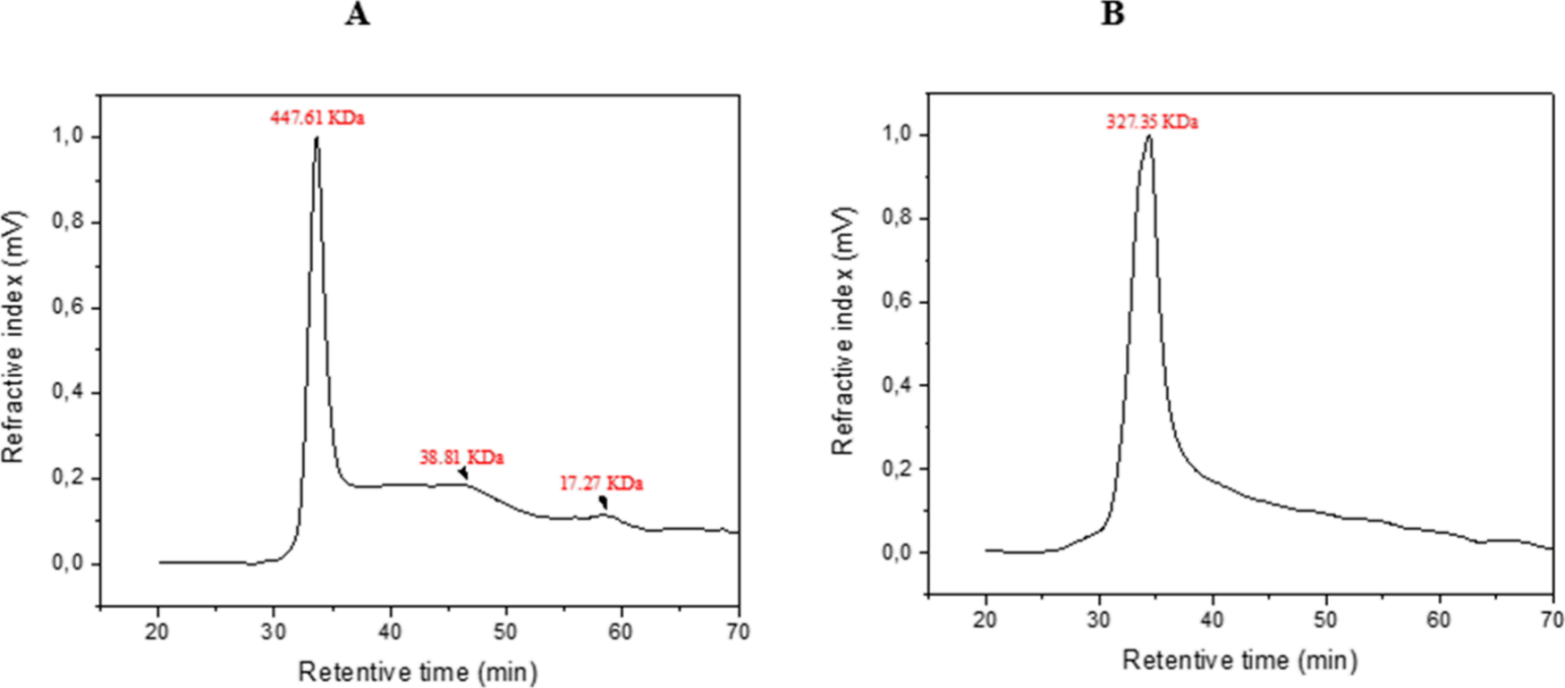
Molecular weight chromatograms of (A) fucoidan and (B) alginate extracted from *M. pyrifera* according to the extraction parameters of method 3 (temperature: 60 °C; pH 3.5; and time 1 h). The molecular weight parameters were calculated based on the calibration curve.

These results demonstrate that, despite variations in the degree of molecular dispersion, both polysaccharides exhibit structural profiles consistent with those previously described for polysaccharides extracted from other brown macroalgae, such as *Saccharina japonica*,^50^ *Cystoseira barbata*,^51^ *Alaria esculenta* and *Saccharina latissima*.^52^

Infrared spectroscopy is based on the qualitative analysis of organic functional chemical groups.^34^ The infrared absorption spectra of fucoidan and alginate exhibited spectra characteristic of polysaccharides extracted from seaweed. Fucoidan showed peaks corresponding to 3364 cm^–1^, attributed to the presence of hydroxyl (OH) groups, and a small signal around 1623 cm^–1^ allowed the identification of the stretching of the acetylated group (C=O).^53^ Absorption peaks were also observed at 1425–1373 cm^–1^, corresponding to the stretching of the carboxyl (COO^–^) groups of hexuronic acid residues, while the absorption spectra at 1222 cm^–1^ refer to the CH_3_ of fucose. In addition, the absorption bands observed at 1026 cm^–1^ are characteristic of the stretching of the C–O–C bond in the pyranosidic ring.^54^ The signals corresponding to 966–822 cm^–1^ refer to the presence of secondary equatorial C–O–S groups at C2 and C4 of the fucopyranose residues, respectively^55^ (Figure 6A).

**Figure 6:**
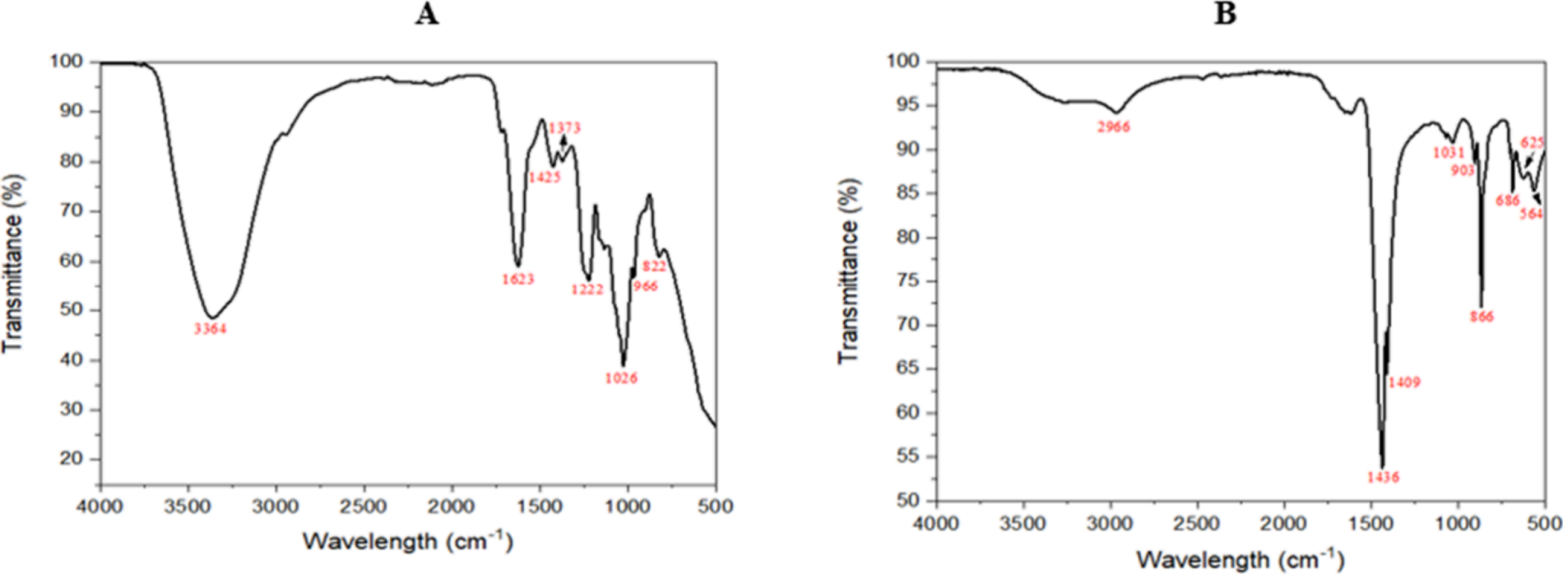
FTIR spectra of (A) fucoidan and (B) alginate extracted from *M. pyrifera* according to the extraction parameters of method 3 (temperature: 60 °C; pH 3.5; and time 1 h) in region from 500 to 4000 cm^–1^ with resolution of 4 cm^–1^.

The functional groups of alginate chemicals were also analyzed by FTIR. The ALG (Figure 6B) showed signals observed at 2966 cm^–1^, with emphasis on O–H and C–H stretching vibrations, respectively.^56^ The band at 1436 cm^–1^ is attributed to asymmetric C–O stretching vibrations, while the band at 1409 cm^–1^ is associated with symmetric C–O vibrations.^57^ The absorption peaks observed at 1031 cm^–1^ are characteristic of alginate and are associated with C–O and C–O–C stretching vibrations, related to the uronic units (M and G residues) of the chain.^58^ Furthermore, the region between 903 and 866 cm^–1^ is attributed to vibrations of the glycosidic backbone and out-of-plane deformations typical of β-(1→4) linkages of uronic sugar rings. The absorption bands at 686, 625, and 564 cm^–1^ are associated with vibrations and bending of the C–O and C–C linkages, contributing to the spectral signature of alginate in the 1000–500 cm^–1^ range.^57^ FTIR analysis revealed the absence of bands in the 1230–1280 cm^–1^ region, attributed to the S = O stretching of sulfate ester groups, confirming the high purity of the extracted alginate, without contamination by sulfated polysaccharides, such as fucoidan.^56^

The monosaccharide composition of fucoidan and alginate extracted from *M. pyrifera* was determined by HPAEC-PAD analysis after hydrolysis with sulfuric acid. The curves and linear equations of the mannitol, fucose, arabinose, rhamnose, galactose, glucose, xylose, mannose, glucuronic acid, guluronic acid, and manuronic acid standards were obtained by integrating the areas multiplied by the concentrations of the standards (Figure S2: Supporting Information). The monosaccharide analysis revealed the presence of fucose (40.07%) as the main sugar constituent of fucoidan, followed by significant proportions of glucuronic acid (20.69%), galactose (17.75%), and glucose (17.73%) and lower levels of arabinose (2.55%) and mannitol (1.19%) (Table 5), justifying the heteropolysaccharide nature of fucoidan.^59^ Alginate had higher proportions of manuronic acid (61.05%), accompanied by relevant amounts of guluronic acid (38.94%) (Table 5), indicating an M/G ratio greater than 1, characteristic of alginates rich in M blocks. This structural profile is generally associated with more flexible chains, lower conformational rigidity, and the formation of more elastic and less mechanically resistant gels, in addition to presenting lower affinity for divalent ions, such as Ca^2+^, when compared to alginates rich in G blocks.^60^

**Table 5:** Analysis of the Monosaccharide Composition of Fucoidan and Alginate Extracted from *M. pyrifera* ^a^

**Monosaccharide (%)**	**FUC**
Mannitol	1.19
Fucose	40.07
Arabinose	2.55
Rhamnose	ND
Galactose	17.75
Glucose	17.73
Xylose	ND
Mannose	ND
Glucuronic acid	20.69

**Monosaccharide (%)**	**ALG**
Mannuronic acid	61.05
Guluronic acid	38.94

a Monosaccharide composition of fucoidan and alginate extracted from *M. pyrifera* obtained by high-performance anion-exchange chromatography with pulsed amperometric detection (HPAEC-PAD) using the ICS5000+ system (Dionex, Sunnyvale, USA) on a CarboPacPA1 column (4 × 250 mm) and a guard column (2 × 50 mm). Standard monosaccharides based on the percentage relative to the total amount of monosaccharide residues: mannitol, fucose, arabinose, rhamnose, galactose, glucose, xylose, mannose, glucuronic acid, guluronic acid, and mannuronic acid.

Fucoidans and alginates are polysaccharides typical of brown macroalgae, with chemical compositions based on fucose and uronic acids, respectively. Fucoidan refers to a complex group of sulfated heteropolysaccharides, characterized by a high proportion of fucose (usually >40%). However, the monosaccharide composition of fucoidans is variable, being related to factors such as species, collection site, seasonality, and environmental conditions, which supports the structurally heterogeneous nature of these polysaccharides.^8^ Previously reported data for *M. pyrifera* and other species of the genus *Macrocystis* show that fucoidans have fucose as the major sugar (≈35–60%), accompanied by variable proportions of galactose (≈5–20%), glucose (≈5–25%), mannose (≈2–10%), arabinose, and xylose in lower amounts (<5%), as well as relevant amounts of uronic acids, which can vary between 10 and 30%, corroborating the wide compositional diversity of fucoidans extracted from these macroalgae.^59,61–63^

Alginate is an anionic polysaccharide composed of repeating units of β-d-mannuronic acid (M) and α-l-guluronic acid (G), organized along the polymer chain into homopolymeric blocks of M (M blocks), homopolymeric blocks of G (G blocks), and alternating MG regions.^9^ The relative proportion between these units, expressed as the M/G ratio, is a key structural parameter influencing the physicochemical and functional properties of alginate. Alginates with lower M/G values (M/G < 1), corresponding to a higher proportion of G residues, exhibit greater conformational rigidity and higher affinity for divalent cations, especially Ca^2+^, due to the formation of egg-box-like structures, resulting in more rigid and stable gels. In contrast, alginates with higher M/G values (M/G > 1) tend to form more flexible and elastic gels with lower mechanical strength.^64^

NMR spectroscopy is a useful method for elucidating the composition of monosaccharides, α/β anomeric configurations, the position of methyl and acetyl groups, and determining the types of glycosidic bonds in polysaccharides.^34^ The HSQC spectrum assignments of fucoidan and alginate are shown in Figure 8. Based on the NMR signals, the anomeric protons of fucoidan were identified in the range of δH 4.32–5.30 ppm (H-1), while the anomeric carbons were observed between δC 97.79–103.01 ppm (C-1) (Figure 7a; Figure S3 Supporting Information). Analysis of the HSQC spectrum, combined with an extensive literature review, allowed the identification of nine spin systems attributed to the following glycosidic residues: (A) →2,3)-α-l-Fuc*p*-(1→, (B) →3)-α-l-Fuc*p*-(2-SO_3_^–^)-(1→, (C) α-l-Fuc*p*-(1→2)→, (D) →4)-α-l-Fuc*p*-(2-SO_4_^2–^)- (1→3)→, (E) →3)-α-l-Fuc*p*-(1→, (F) →3)-β-d-GlcA*p*-(1→, (G) →6)-β-d-Gal*p*-(1→, (H) β-d-Gal*p*-(1→6)→ and α-d-Glc*p*-(1→ (Table 6; Figure 8a).

**Figure 7:**
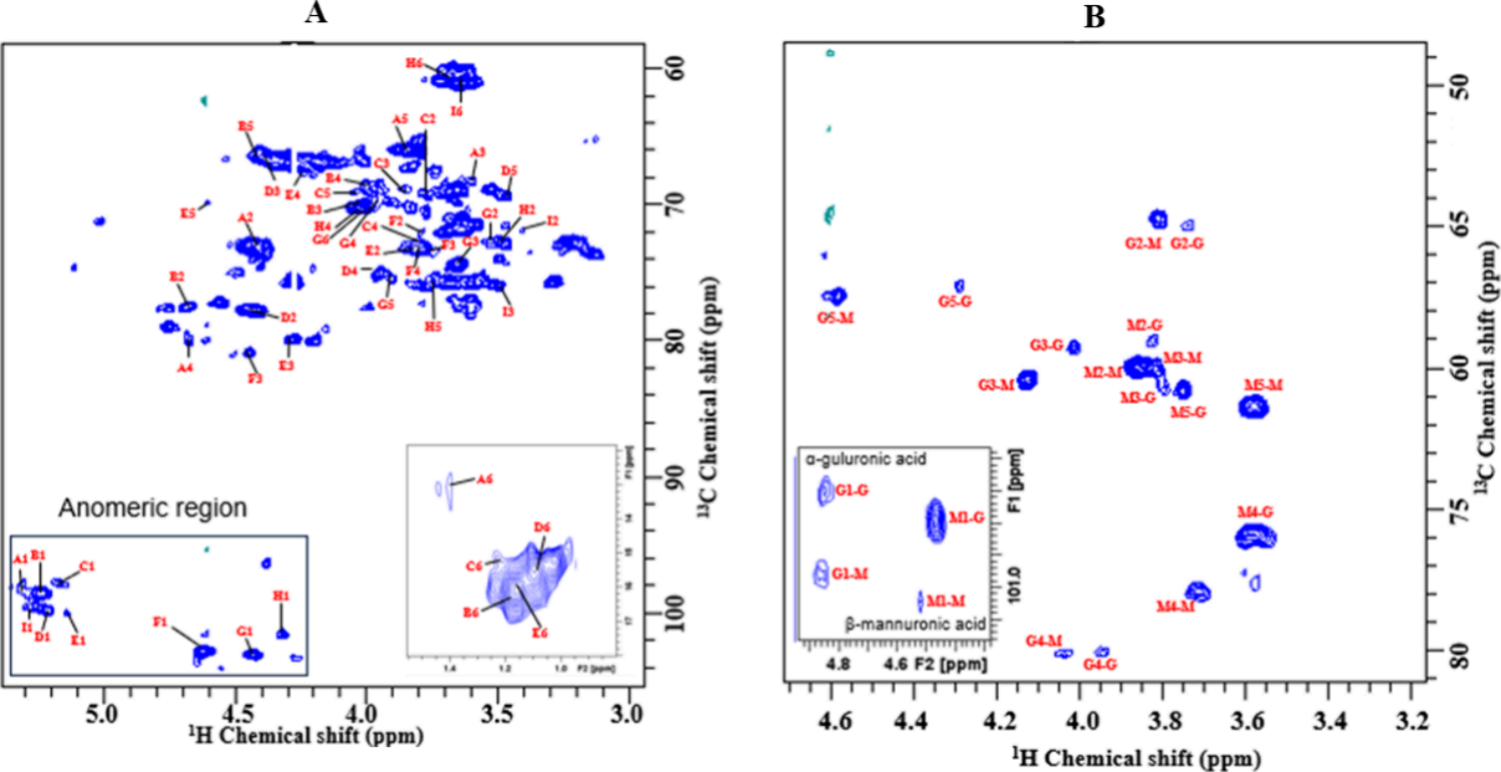
Structural analysis of polysaccharides extracted from *M. pyrifera* by HSQC NMR recorded at 60 °C on a Bruker DRX spectrometer at 600 MHz: (A) fucoidan spectrum and (B) alginate.

**Table 6:** HSQC (^1^H -^13^C) NMR Data of Fucoidan Extracted from *M. pyrifera* ^a^

		**Chemical shifts (ppm)^b^**					
**Residue**	**Sugar linkage**	H-1/C-1	H-2/C-2	H-3/C-3	H-4/C-4	H-5/C-5	H-6/C-6
A	→2,3)-α-l-Fuc*p*-(1→	5.30/97.86	4.41/72.8	3.61/68.30	4.68/79.70	3.84/65.90	1.40/12.92
B	→ 3)-α-l-Fuc*p*-(2-SO_3_^–^)-(1→	5.24/98.51	4.71/77.60	4.01/70.20	3.98/68.30	4.43/66.30	1.16/16.08
C	α-l-Fuc*p*-(1→ 2)→	5.17/97.79	3.77/69.27	3.86/68.77	3.81/73.21	4.04/68.92	1.22/15.5
D	→ 4)-α-l-Fuc*p*-(2-SO_4_^2–^)-(1→ 3)→	5.23/99.49	4.43/77.71	4.33/67.10	4.03/74.00	3.47/69.40	1.10/15.57
E	→ 3)-α-l-Fuc*p*-(1→	5.13/99.97	3.80/72.85	4.31/79.90	4.19/67.80	4.61/69.60	1.16/16.05
F	→3)-β-d-GlcA*p*-(1→	4.62/102.78	3.76/71.7	4.47/81.00	3.84/72.90		
G	→ 6)-β-d-Gal*p*-(1→	4.43/103.01	3.52/72.50	3.66/73.80	3.96/69.50	3.84/75.20	4.03/70.07
H	β-d-Gal*p*-(1→6)→	4.32/101.61	3.54/72.70	3.76/73.20	3.98/69.70	3.76/75.70	3.69/60.47
I	α-d-Glc*p*-(1→	5.26/99.47	3.39/71.80	3.50/75.70			3.63/61.00

a The data were obtained from a combination of ^1^H and HSQC (^1^H/^13^C) spectra at 600 MHz.

b The chemical shifts are based on tetramethylsilane—TMS.

**Figure 8:**
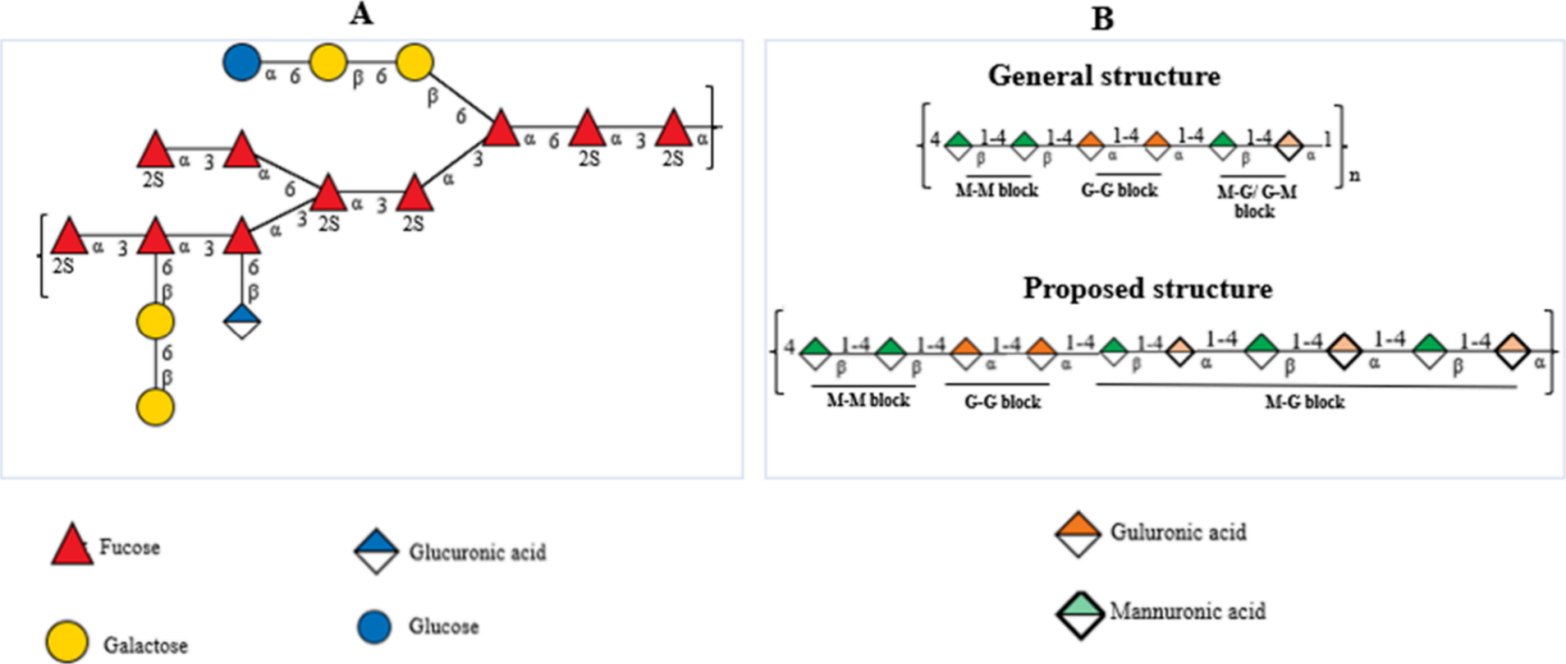
Schematic structure of the (A) fucoidan and (B) alginate extracted from *M. pyrifera* obtained from the structure editor of the Carbohydrate Structure Database using the Symbol Nomenclature for Glycans—CSDB/SNFG.

Residues B, D, and E were assigned to the main chain of fucoidan based on the chemical shift values observed in the HSQC spectrum. Residue B showed signals at δH/δC 5.24/98.51 (H-1/C-1), 4.71/77.60 (H-2/C-2), 4.01/70. Twenty (H-3/C-3), 3.98/68.30 (H-4/C-4), 4.43/66.30 (H-5/C-5), and 1.16/16.08 ppm (H-6/C-6). Residue D showed shifts at 5.23/99.49, 4.43/77.71, 4.33/67.10, 4.03/74.00, 3.47/69.40, and 1.10/15.57 ppm, while residue E showed signals at 5.13/99.97, 3.80/72.85, 4.31/79.90, 4.19/67.80, 4.61/69.60, and 1.16/16.05 ppm (Table 6). The anomeric pattern close to δH 5.0–5.3 ppm and δC ∼ 98–100 ppm confirms the α-l configuration of the fucopyranose residues, while the deshielded shifts at C-2 and/or C-4 indicate substitution by sulfate groups.^65,66^ Together, these data allowed the residues to be assigned as (B) →3)-α-l-Fucp-(2-SO_3_^–^)-(1→, (D) →4)-α-l-Fucp-(2-SO_4_^2–^)-(1→3)→ and (E) →3)-α-l-Fucp-(1→, corroborating the presence of a main chain predominantly formed by sulfated fucose units, a structural feature widely described for fucoidans.^65^

Residues A and C were assigned to α-l-fucopyranose units with different substitution patterns, indicating more complex structural regions along the polymer. Residue A, identified as →2,3)-α-l-Fuc*p*-(1→ and with signals at δH/ δC 5.30/97.86, 4.41/72.8, 3.61/68.30, 4.68/79.70, 3.84/65.90, and 1.40/12.92 ppm suggests that the anomeric shift is consistent with the α-l configuration of fucose, while the low frequency shift observed at C-2 and C-3 indicates substitution at these positions, suggesting a branching point resulting from glycosylation at the O-2 and O-3 hydroxyl groups, a characteristic associated with the highly branched structure of heterofucans.^67^ Residue C, designated as α-l-Fuc*p*-(1→2)→, indicates a fucose unit involved in glycosidic bonds at the O-2 position, possibly acting in the bond between the main chain and side chains.^66^

In addition to fucose units, other monosaccharides were identified that corroborate the heteropolysaccharide nature of fucoidan. The F residue was attributed to →3)-β-d-glucuronic acid-(1→ based on the chemical shifts observed in δH/ δC 4.62/102.78 (H-1/C-1), 3.76/71.70 (H-2/C-2), 4.47/81.00 (H-3/C-3), and 3.84/72.90 (H-4/C-4) ppm. The anomeric signal at δC 102.78 ppm, associated with δH less than 5.0 ppm, is characteristic of the β configuration in pyran sugars, while the low frequency shift of C-3 (∼81 ppm) indicates glycosidic substitution at this position, confirming the (→3) linkage pattern.^68^ The residues G (→6)-β-d-Gal*p*-(1→) and H (β-d-Gal*p*-(1→6)→) correspond to galactopyranose units involved in (1→6) bonds, often related to the formation of flexible side chains.^66^ These values are within the typical range described for uronic acid and galactan residues associated with sulfated fucose units, whose presence contributes to the anionic character, solubility, and biological properties of fucoidan.^66,68^

The presence of the terminal residue α-d-Glc*p*-(1→ was identified in the HSQC spectrum, indicating the occurrence of nonsubstituted glucose units at the extremities of the polysaccharide chain. The absence of substitution positions suggests that this residue is not involved in further glycosidic linkages, characterizing it as a terminal unit, possibly located at the end of side chains or minor branches.^69,70^ The anomeric signal observed at δH/δC 5.26/99.47 ppm, together with the correlations at 3.39/71.80 ppm (H-2/C-2), 3.50/75.70 ppm (H-3/C-3), and 3.63/61.00 ppm (H-6/C-6), is consistent with the α-D configuration of glucopyranose.^69^ Signals corresponding to H-4/C-4 and H-5/C-5 were partially overlapped, preventing unambiguous assignment.

The HSQC results combined with monosaccharide composition analysis primarily reveal the primary structure of the polymer, including glycosidic linkage patterns and substitution positions, whereas the compositional analysis reflects the pool of monosaccharides released after hydrolysis, which may include coextracted polysaccharides and differences in hydrolytic susceptibility.^71^ Recent studies have shown that fucoidans from *M. pyrifera* exhibit variable composition, typically rich in fucose, but also containing galactose, glucuronic acid, and minor amounts of neutral sugars, whose distribution may be influenced by biomass origin and extraction conditions.^59^ In this context, the relatively high glucose content may be associated with the coextraction of glucans such as laminarin. The proportions of fucose, glucuronic acid, galactose, and the identification of a terminal α-d-Glcp-(1→ residue in the HSQC spectrum further support the heteropolysaccharidic nature of the fucoidan.^8,72^

Structural analysis by HSQC NMR and monosaccharide composition showed that fucoidan consists of a backbone of sulfated α-l-fucopyranose residues (→3)-α-l-Fuc*p*-(2-SO_3_^–^)-(1→, →4)-α-l-Fuc*p*-(2-SO_4_^2–^)-(1→3)→ and →3)-α-l-Fuc*p*-(1→), predominantly linked by (1→3) and (1→4) glycosidic linkages and partially sulfated at C-2, with branching occurring at the O-2 and O-3 positions. The side chains include β-d-glucuronic acid and β-d-galactopyranose residues, characterizing the polymer as a heterofucan (Figure 8A). Furthermore, the detection of a terminal α-d-Glc*p*-(1→) residue indicates the presence of terminal nonreducing glucose units, possibly associated with smaller side chains or coextracted glucans, further contributing to the structural complexity of the polysaccharide. (Figure 8A).

The HSQC spectrum of alginate extracted from *M. pyrifera* is shown in Figure 7B, allowing the identification of the main characteristic dyads of the copolymer formed by β-d-mannuronic acid (Man*p*A) and α-l-guluronic acid (Gul*p*A) residues. The correlations observed in the spectra enabled the assignment of the homopolymeric blocks M–M (→4)-β-d-Man*p*A-(1→4)-β-d-Man*p*A-(1→) and G–G (→4)-α-l-Gul*p*A-(1→4)-α -l-Gul*p*A-(1→), as well as the heteropolymeric sequences M–G (→4)-β-d-Man*p*A-(1→4)-α-l-Gul*p*A-(1→) and G–M (→4)-α-l-Gul*p*A-(1→4)-β-d-Man*p*A-(1→). The anomeric chemical shifts were consistent with values described in the literature for macroalgae alginates, with H-1/C-1 signals from manuronic residues distributed in the range of 4.46–4.51 ppm and 99.93–101. Twenty-five ppm, while the guluronic residues showed signals between 4.85–4.86 ppm and 99.49–100.77 ppm (Table 7).^73,74^

**Table 7:** HSQC (^1^H -^13^C) NMR Analyses of Alginate Extracted from *M. pyrifera* ^a^

			**Chemical shifts (ppm)^b^**				
**Residue**	**Abbreviation**	**Sugar linkage**	H-1/C-1	H-2/C-2	H-3/C-3	H-4/C-4	H-5/C-5
A	M–M	→4)-β-d-Man*p*A-(1→4)-β-d-ManpA-(1→	4.51/101.25	3.86/69.96	3.81/69.89	3.71/77.86	3.58/71.2
B	M–G	→4)-β-d-Man*p*A-(1→4)-α-l-GulpA-(1→	4.46/99.93	3.82/69.07	3.79/70.55	3.57/75.90	3.75/70.87
C	G–G	→4)-α-l-Gul*p*A-(1→4)-α-l-GulpA-(1→	4.85/99.49	3.73/64.93	4.01/69.24	3.94/80.04	4.29/67.08
D	G–M	→4)-α-l-Gul*p*A-(1→4)-β-d-Man*p*A-(1→	4.86/100.77	3.80/64.67	4.12/70.42	4.03/79.95	4.58/67.48

a The data were obtained from a combination of HSQC (^1^H/^13^C) spectra at 600 MHz.

b The chemical shifts are based on tetramethylsilane—TMS.

The chemical shifts of the H-2/C-2, H-3/C-3, H-4/C-4, and H-5/C-5 correlations confirmed the structural heterogeneity of alginate and were consistent with values described for polymers consisting of Man*p*A and Gul*p*A residues (Table 7). For the M–M dyad, signals were observed at 3.86/69.96 ppm (H-2/C-2), 3.81/69.89 ppm (H-3/C-3), 3.71/77.86 ppm (H-4/C-4), and 3.58/71.20 ppm (H-5/C-5). The M–G sequence showed correlations at 3.82/69.07, 3.79/70.55, 3.57/75.90, and 3.75/70.87 ppm for H-2/C-2 to H-5/C-5, respectively. For the homopolymeric G–G block, the shifts were recorded at 3.73/64.93, 4.01/69.24, 3.94/80.04, and 4.29/67.08 ppm, while the G–M dyad showed signals at 3.80/64.67, 4.12/70.42, 4.03/79.95, and 4.58/67.48 ppm.^74,75^ The shifts observed for C-4 in the guluronic residues reflect the characteristic conformation of these monomers, while the variations in carbons C-3 and C-5 show the influence of the adjacent residue in the chain with homopolymeric blocks and alternating sequences.^73,76^

Quantitative analysis of the integrals (Table 8) revealed a predominance of manuronic units, corresponding to 60.28% of the total composition, while guluronic residues represented 39.72%, which indicated agreement with the monosaccharide composition determined for alginate with 61.05% manuronic acid and 38.94% guluronic acid. In addition, the M/G ratio of 1.52 indicates manuronic predominance. Among the dyads, the heteropolymeric M–G block contributed the most (52.11%), followed by the G–G (23.12%) and G–M (16.60%) blocks, while the M–M fraction was less abundant (8.16%). This pattern indicates a copolymer with a significant distribution of alternating sequences, suggesting a less rigid chain organization when compared to alginates rich in guluronic blocks.^9^

**Table 8:** Integral and Relative Analysis of Mannuronic (M) Acid and Guluronic acid (G) Blocks in Alginate Extracted from *M. pyrifera* Obtained from HSQC (^1^H–^13^C) NMR

**Unit**	**Absolute integral**	**%**
M–M	56873.00	8.16
M–G	362790.00	52.11
Total mannuronic acid	419663.00	60.28
G–G	161000.00	23.12
G–M	115580.00	16.60
Total guluronic acid	276580.00	39.72
Manuronic/guluronic	1.52	

From a structural and functional point of view, the predominance of manuronic residues associated with the high proportion of heteropolymeric dyads suggests greater conformational flexibility and potential for the formation of more elastic gels, characteristics related to alginates with high M/G values.^9^ In contrast, the smaller fraction of M–M blocks indicates a reduced occurrence of highly ordered regions, typically responsible for the formation of cooperative binding zones with divalent cations.^77^ Together, the results confirm that the alginate analyzed has a typical MM, GG, and MG or GM copolymer architecture (Figure 8B), providing structural evidence for applications with rheological properties.

## 4 Conclusions

This study demonstrated that the optimized sequential extraction strategy is an efficient and sustainable approach for the full valorization of *M. pyrifera*. The high carbohydrate content of the biomass, combined with its low levels of lipids, proteins, and phenolic compounds, confirmed its suitability as a raw material for polysaccharide recovery. The application of factorial design allowed for the identification of optimal extraction conditions, resulting in efficient isolation of fucoidan and alginate.

In sequential extraction, the biostimulant obtained by osmotic treatment stands out for its initial use of biomass, highlighting the efficiency of osmotic treatment as a gentle and sustainable strategy for obtaining soluble bioactive compounds. Chemical, chromatographic, and spectroscopic analyses confirmed the typical structural characteristics of polysaccharides. Fucoidans have a main chain composed predominantly of α-l-fucosopyranose units linked by glycosidic bonds (1→3) and (1→4), partially sulfated at C-2, with branched residues of β-d-glucuronic acid and β-d-galactose, consistent with the bioactive fucoidans reported in the literature. Alginate was a distributed in blocks of manuronic and guluronic acids, with a predominance of MG blocks, indicating structural characteristics associated with favorable rheological and functional properties.

Overall, the results highlight that the proposed sequential extraction process allows for the integrated recovery of multiple high value-added products, including biostimulant, fucoidan, and alginate, maximizing biomass utilization. This approach strengthens the potential of *M. pyrifera* as a renewable marine resource for biotechnological applications and provides a scalable framework for the development of sustainable biorefineries.

## Supplementary Material



## Notes

Factor coding and factorial design matrix; elemental composition of biostimulants; molecular weight calibration curve and molecular weight data of fucoidan and alginate; and monosaccharide standard chromatogram and ^1^H NMR spectrum of fucoidan (PDF)
