# Supplementary material for: Optimization of the Sequential Extraction Process of Compounds for the Integral Valorization of Macrocystis pyrifera
Source: ACS Omega. 2026 Jul 1;11(27):40402–16. doi: 10.1021/acsomega.6c03104 (PMC13382842; doi:10.1021/acsomega.6c03104)
Supplement: Supplementary file 1 [file ao6c03104_si_001.pdf]

Optimization of the sequential extraction process of compounds for the integral valorization of *Macrocystis pyrifera*

Francisca Crislândia Oliveira Silva[a], Giovanna de Farias Barreto[a], Thiago Barbosa Cahú[b], Francisco Felipe Bezerra[b], Lidilhone Hamerski[c], Javier Infante Rosselot[d], Mauro Sérgio Gonçalves Pavão[a]

[a] Federal University of Rio de Janeiro, Institute of Medical Biochemistry, Laboratory of Biochemistry and Cell Biology of Glycoconjugates, Street Prof°. Rodolpho P. Rocco, 255, Rio de Janeiro, 21941-913, Brazil.
[b] Federal University of Rio de Janeiro, Institute of Medical Biochemistry, Connective Tissue Laboratory, Street Prof°. Rodolpho P. Rocco, 255, Rio de Janeiro, 21941-913, Brazil.
[c] Federal University of Rio de Janeiro, Natural Products Research Institute, Carlos Chagas Filho Street 373, Rio de Janeiro, 21941-902. Brazil.
[d] Ocean Rainforest, Inc., 1117 State Street, Santa Barbara, CA 93101, USA.

Corresponding author

E-mail addresses: mpavao@hucff.ufrj.br
Phone: +55 (21) 97176-8963. Fax: +55 (21) 3938-2481

SUPPLEMENTARY MATERIAL

Table S1: Coding of extraction to Levels (2^3).

Factors	Levels	
	**-1**	**+1**
Temperature	60	80
pH	3.0	3.5
Time	1	2

Table S2: Factorial Planning Matrix.

Extraction	Temperature (°C)	pH	Time (Hours)
1	-	-	-
2	-	-	+
3	-	+	-
4	-	+	+
5	+	-	-
6	+	-	+
7	+	+	-
8	+	+	+

Table S3: Elemental analysis of macronutrients and micronutrients of biostimulants: *M. pyrifera* biostimulant obtained by osmotic shock treatment (MPBIO-OST) and *M. pyrifera* biostimulant obtained by heat treatment (MPBIO-HT). Different letters indicate statistically significant differences between the biostimulant extracts. Differences were considered statistically significant at $p < 0.05$.

	**MPBIO-OST**	**MPBIO-HT**
**Macronutrients (mg/L)**		
Nitrogen (N)	190±2.828[a]	190±2.828[a]
Phosphorus (P)	20±1.414[a]	20±1.414[a]
Potassium (K)	1340±1.4114[a]	1350±1.414[a]
Calcium (Ca)	470±1.442[a]	430±1.442[a]
Magnesium (Mg)	350±1.142[a]	330±1.142[a]
Sulfur – as sulfate ($S–SO_4^{2-}$)	190±7.071[a]	140±7.071[b]
**Micronutrients (mg/L)**		
Iron (Fe)	0.53±0.014[a]	0.53±0.014[a]
Manganese (Mn)	0.06±0.014[a]	0.04±0.014[a]
Copper (Cu)	0.14±0.014[a]	0.10±0.014[a]
Zinc (Zn)	0.03±0.014[a]	0.01±0.007[a]
Boron (B)	6.10±0.141[a]	5.14±0.212[b]

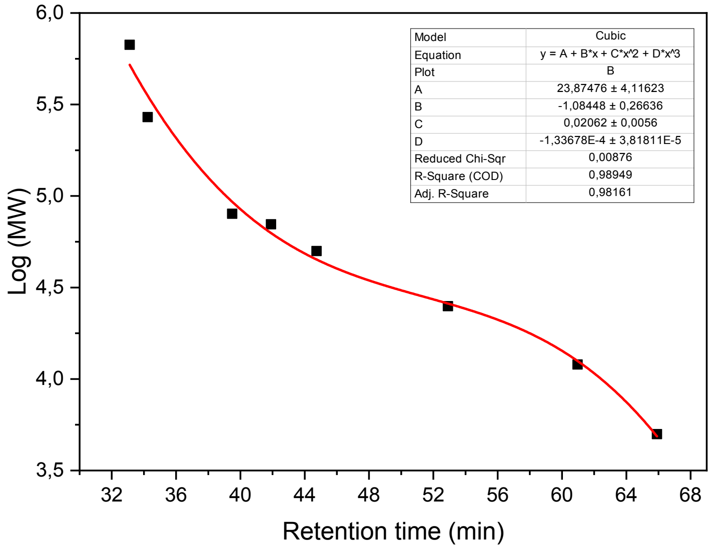

Figure S1: Calibration curve with dextran standards (5 kDa, 12 kDa, 25 kDa, 50 kDa, 70 kDa, 80 kDa, 270 kDa, and 670 kDa) by gel permeation chromatography (GPC) analysis.

Table S4: Molecular weight of fucoidan and alginate extracted from *M. pyrifera* according to the extraction parameters of method 3 (temperature: 60 °C; pH 3.5 and time 1 h). The molecular weight parameters were calculated based on the calibration curve.

**Parameters**		**FUC**		**ALG**
**Retention time (min)**	33.55	46.57	58.49	34.49167
**Molecular weigh (Da)**	447610.61	38805.65	17269.97	327348.48
**Molecular weigh (KDa)**	447.61	38.81	17.27	327.35
**Molecular weigh (Log)**	5.65	4.59	4.24	5.52

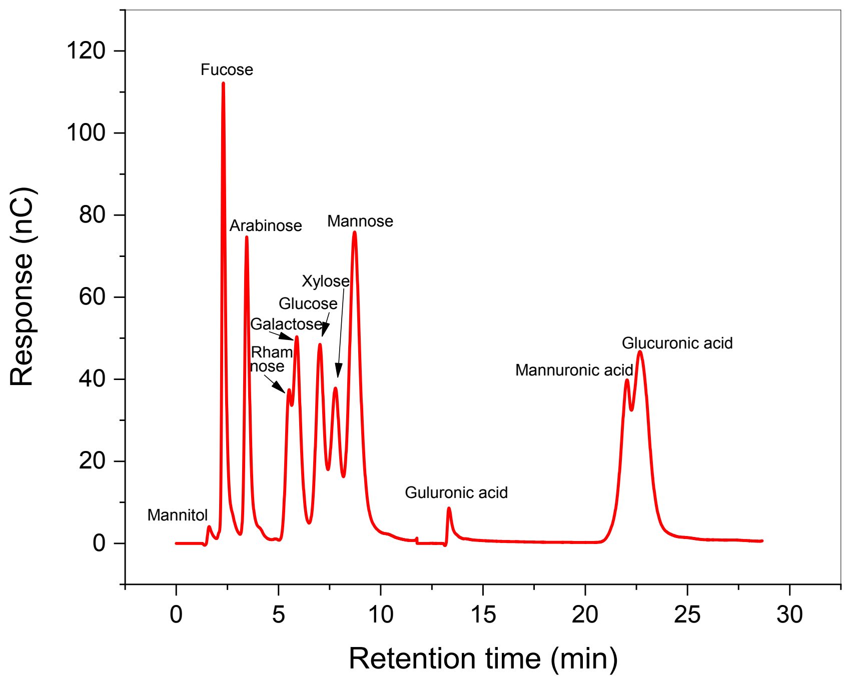

Figure S2: Chromatogram of the standard curve of the monosaccharide composition of fucoidan and alginate extracted from *M. pyrifera* obtained by high-performance anion-exchange chromatography with pulsed amperometric detection (HPAEC-PAD) using the ICS5000+ system (Dionex, Sunnyvale, USA) on a CarboPac PA1 column (4 × 250 mm) and guard column (2 × 50 mm). Standard monosaccharides based on the percentage relative to the total amount of monosaccharide residues: mannitol, fucose, arabinose, rhamnose, galactose, glucose, xylose, mannose, glucuronic acid, guluronic acid, mannuronic acid, and glucuronic acid.

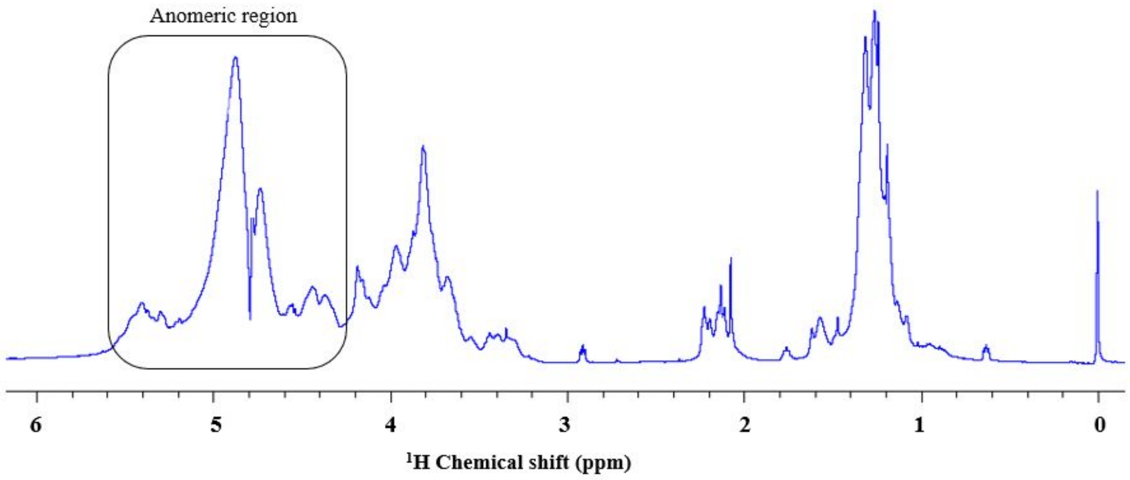

Figure S3: ^{1}H NMR spectra of the fucoidan extracted from *Macrocystis pyrifera* recorded at 60 °C on a Bruker DRX spectrometer at 600 MHz.
